# Carcinome épidermoïde primitif du colon: à propos d’un cas

**DOI:** 10.11604/pamj.2017.27.124.12037

**Published:** 2017-06-15

**Authors:** Sinaa Mohamed

**Affiliations:** 1 Laboratoire d’Anatomie Pathologique, Hôpital Militaire Moulay Ismail, Meknès, Maroc

**Keywords:** Colon, carcinome épidermoïde, pronostic, Colon, squamous cell carcinoma, prognosis

## Abstract

Le carcinome épidermoïde (CE) primitif du côlon est une tumeur exceptionnelle. Moins de 150 cas ont été publiés dans la littérature jusqu'à l'année 2014. Outre sa rareté, il se distingue par son association fréquente avec d'autres néoplasies digestifs. Nous rapportons l'observation d'un patient de 54 ans ayant une CE primitive du côlon. À la lumière de cette observation, on discutera les particularités anatomo-cliniques et thérapeutiques ainsi que les hypothèses étiopathogéniques de cette entité peu commune.

## Abstract (en)

Primary squamous cell carcinoma (SCC) of the colon is an exceptional tumor. Less than 150 cases have been reported in the literature up to the year 2014. In addition to its rarity, it is distinguished by its frequent association with other digestive neoplasias. We report the case of a 54-year old patient with primary SCC of the colon. In the light of this case study, we will discuss the anatomo-clinical and therapeutic features as well as the etiopathogenic assumptions of this unusual entity.

## Introduction

La localisation colique d'un carcinome épidermoïde (CE) primitive est exceptionnelle. L'histogenèse d'une telle tumeur ainsi que son potentiel évolutif soulèvent encore des interrogations [1–3].

## Patient et observation

**Figure 1:**
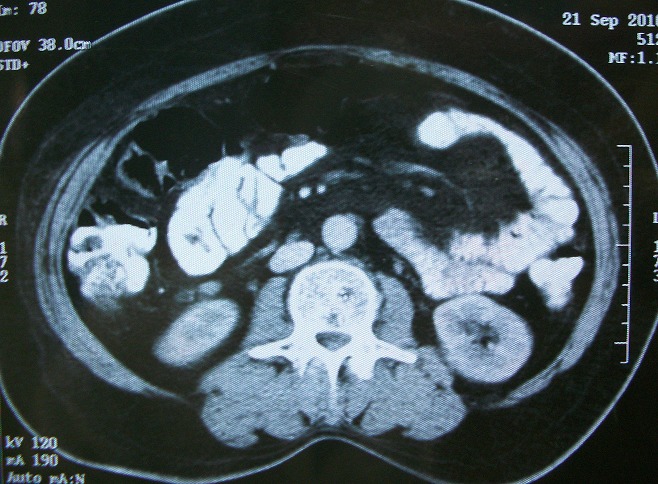
Coupe tomodensitométrique montrant une masse colique droite (flèche)

**Figure 2:**
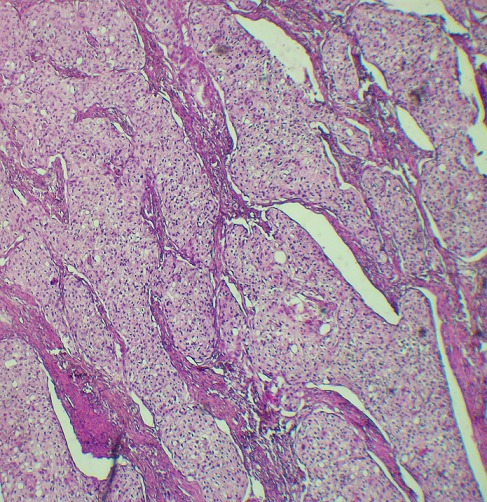
Muqueuse colique siège d’une prolifération carcinomateuse faite d’amas et de massifs, sans structures glandulaires (HEx200)

**Figure 3:**
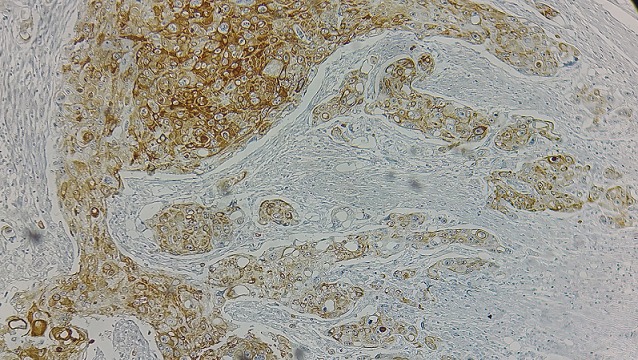
Les cellules tumorales comportant des ponts et des cadres cellulaires, aux cytoplasmes éosinophiles et aux noyaux montrant des atypies modérées à marquées avec des globes cornés(fleche) (HEx200)

**Figure 4:**
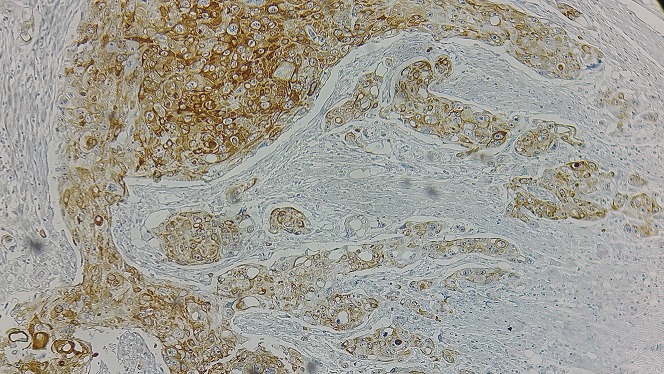
Positivité des cellules tumorales à l’anticorps anti-P63 (x200)

**Figure 5:**
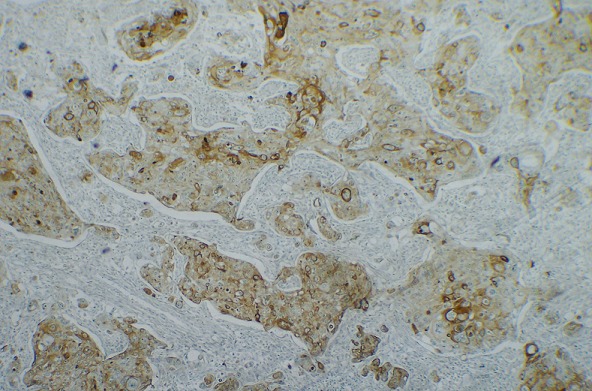
Positivité des cellules tumorales à l’anticorps anti-P40 (x200)

**Figure 6:**
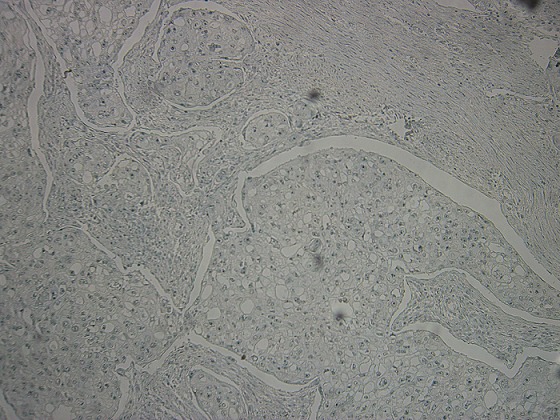
Négativité des cellules tumorales à l’anticorps anti-CK7 (x200)

**Figure 7:**
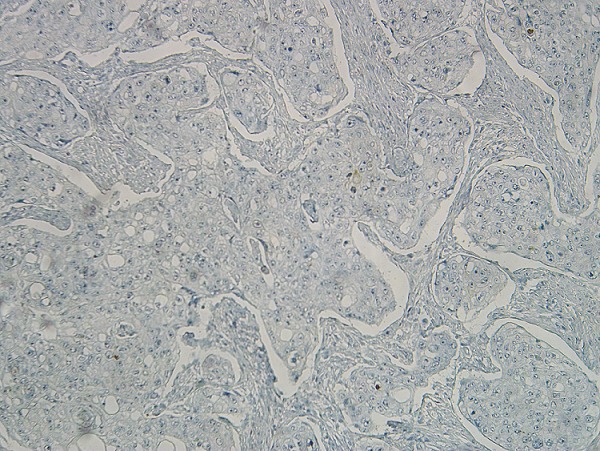
Négativité des cellules tumorales à l’anticorps anti-CK20 (x200)

Un homme âgé de 54 ans, sans antécédents pathologiques notables, présentait des douleurs de l'hypochondre droit associées à une constipation avec des épisodes de méléna et un amaigrissement non chiffré. L'examen physique était normal. La colonoscopie avait montré une tumeur au niveau de l'angle colique droit. La tomodensitométrie abdominale objectivait une masse colique droite d'environ 4,5 cm de grand axe (Figure 1). La fibroscopie œsogastroduodénale était normale. L'étude anatomopathologique de la biopsie a conclu à un processus carcinomateux peu différencié. Une colectomie segmentaire a été réalisée. L'étude macroscopique de la pièce opératoire trouve une tumeur colique bourgeonnante de 3,4x4, 6 cm avec un aspect blanc-grisâtre à la coupe. Après un large échantillonnage de la tumeur, les coupes histologiques montrent une prolifération tumorale carcinomateuse faite d'amas et de boyaux, sans structures glandulaires (Figure 2). Les cellules tumorales sont de grande taille, comportant des ponts et des cadres cellulaires, aux cytoplasmes éosinophiles et aux noyaux montrant des atypies modérées à marquées avec des figures mitotiques et plusieurs globes cornés (Figure 3). Pas vu d'embols vasculaires, ni d'engainements périnerveux. La prolifération arrivait jusqu'à la séreuse. Le complément immunohistochimique a montré une positivité des cellules tumorales aux anticorps anti P63 (Figure 4) et P40 (Figure 5) confirmant leur nature malpighienne. Les CK7 (Figure 6) et CK 20 (Figure 7) étaient négatives. On a conclu à une CE bien différenciée infiltrant et kératinisant du côlon. Le curage ganglionnaire a ramené 14 ganglions indemnes de toute métastase. La tumeur été classé T3N0. L'examen clinique cutanéo-muqueux, ainsi que le bilan d'extension général (scanner thoraco-abdomino pelvienne et IRM encéphalique) n'ont pas montré d'autre localisation secondaire, confirmant ainsi le caractère primitif du CE colique. L'évolution postopératoire était favorable et le patient était suivi en consultation durant 12 mois après son opération, puis il a été perdu de vue.

## Discussion

Le CE primitif colique est extrêmement rare, représentant moins de 0,5 % de l'ensemble des tumeurs colorectales, avec une incidence estimée à 0,1 % [1, 2]. L'étiopathogénie du CE colique est encore imprécise. Il pourrait naître d'une cellule souche multipotente ou se développer sur une métaplasie malpighienne secondaire à une irritation chronique [2–4]. En faveur de cette seconde hypothèse, l'association fréquente du CE colique à des colites inflammatoires chroniques, en particulier la rectocolite ulcérohémorragique, l'incidence relative de cette association étant de 1,7 % alors qu'elle est seulement de 0,25à 0,5‰ dans la population générale [5]. Des colites parasitaires telles que l'amibiase ou la schistosomiase ainsi que des fistules colocutanées chroniques également décrites pourraient être à l'origine de la métaplasie malpighienne [5, 6]. Le CE colique survient en moyenne vers la cinquième décennie avec une prédominance masculine (sex-ratio : 2) [6, 7]. Il siège avec prédilection au niveau du cæcum et du côlon droit [7], comme c'est le cas chez notre patient, beaucoup plus rarement au niveau du côlon gauche. Sur le plan clinique, la symptomatologie est superposable à celle des adénocarcinomes [2, 4], avec un délai diagnostique variable entre six semaines et 12 mois [6]. Des métastases sont parfois révélatrices ; elles sont hépatiques,ou pulmonaires [7, 8]. Les critères diagnostiques du CE colique sont stricts. En effet, la tumeur doit siéger 7 cm au moins au-dessus de la ligne anopérinéale, excluant ainsi tous les CE du canal anal et du bas rectum. Sur le plan histologique, il faut éliminer un carcinome adénosquameux, entité également rare associant deux contingents distincts plus au moins intriqués, glandulaires et malpighiens. Pour cela, la tumeur doit être largement échantillonnée. Enfin, avant de retenir le caractère primitif de la tumeur, il faut rechercher une tumeur épidermoïde dans d'autres organes pouvant être à l'origine de métastases digestives [1, 2, 8, 9]. Dans 10 % des cas, le CE colique est associé à une tumeur digestive, le plus souvent un adénocarcinome synchrone [9, 10]. Il peut également s'associer à des cancers extradigestifs ; des cas de carcinomes ovarien, endométrial, prostatique et mammaire ont été rapportés [10, 11]. Les résultats du traitement adjuvant sont aléatoires. L'efficacité de la radiothérapie associée ou non à une chimiothérapie n'a pas été établie vu la rareté de la tumeur [1, 2, 10]. Le CE colique est de mauvais pronostic par rapport à son homologue glandulaire. le décès survient avant la première année dans 52 % des cas [10]. Certains facteurs seraient associés à une évolution péjorative : la localisation gauche de la tumeur, le caractère ulcéré de la lésion, les métastases ganglionnaires, le degré de la différenciation de la tumeur (peu différenciée et indifférenciée) et le stade IV de TNM [1, 10].

## Conclusion

Le CE primitif colique est une tumeur exceptionnelle d'étiopathogénie imprécise. Son carcinogenèse est toujours mal élucidé. Le pronostic est plus réservé que celui de l'adénocarcinome habituel.

## Conflits d’intérêts

Les auteurs ne déclarent aucun conflit d'intérêt.
